# Vessel state and immune infiltration of the angiogenesis subgroup and construction of a prediction model in osteosarcoma

**DOI:** 10.3389/fimmu.2022.992266

**Published:** 2022-11-02

**Authors:** Jintao Wu, Zhijian Jin, Jianwei Lin, Yucheng Fu, Jun Wang, Yuhui Shen

**Affiliations:** ^1^ Department of Orthopaedics, Ruijin Hospital, Shanghai Jiao Tong University School of Medicine, Shanghai, China; ^2^ Department of General Surgery, Ruijin Hospital, Shanghai Jiao Tong University School of Medicine, Shanghai, China

**Keywords:** angiogenesis, vessel state, immune infiltration, prognosis, osteosarcoma

## Abstract

Angiogenesis has been recognized as a pivotal contributor to tumorigenesis and progression. However, the role of angiogenesis-related genes (ARGs) in vessel state, immune infiltration, and prognosis remains unknown in osteosarcoma (OS). Bulk RNA sequencing data of osteosarcoma patients were obtained from the Therapeutically Applicable Research to Generate Effective Treatments (TARGET) database, and patients were divided into two angiogenesis subgroups according to the expression of ARGs. We compared their vessel state and used two independent algorithms to evaluate the tumor microenvironment (TME) in the two subgroups. Furthermore, hub genes of differentially expressed genes (DEGs) in the two subgroups were selected to perform LASSO regression and multivariate Cox stepwise regression, and two prognostic hub genes were found. An ARG_score based on prognostic hub genes was calculated and proved to be reliable in the overall survival prediction in OS patients. Furthermore, the ARG_score was significantly associated with ARGs, immune infiltration, response to immunotherapy, and drug sensitivity. To make our prediction model perform well, clinical features were added and a highly accurate interactive nomogram was constructed. Immunohistochemistry and qRT-PCR were utilized to verify the expression of prognostic hub genes. GSE21257 from the Gene Expression Omnibus (GEO) database was used as a validation dataset to verify its robustness. In conclusion, our comprehensive analysis of angiogenesis subgroups in OS illustrated that angiogenesis may lead to different vessel states and further affect immune infiltration and prognosis of OS patients. Our findings may bring a novel perspective for the immunotherapy strategies for OS patients.

## Introduction

Osteosarcoma (OS) is the most common primary malignant bone tumor and mainly occurs in children and adolescents (1). Over decades, the prognosis of OS patients had reached a discouraging plateau due to its high heterogeneity (2). Recently, immunotherapy has become a promising therapy that has proved to be efficient in many tumors (3–5). Immunotherapy strategies mainly include immune checkpoint inhibitors (ICIs), chimeric antigen receptor (CAR) T cells, tumor vaccines, and so on (6). ICIs, involving inhibitors of PD-1, PD-L1, and CTLA-4, can reverse the state of immunosuppression and mobilize the immune cells to attack the tumor instead of targeting the tumor itself (7). Despite many advantages, immunotherapy can only benefit a small group of patients (8, 9). More potential mechanisms which influence the effect of immunotherapy need to be explored for the identification of patients with immunosensitivity.

Angiogenesis, which refers to the process of sprouting, is regulated by a large number of pro- and anti-angiogenic molecules (10, 11). Following the formation of primary vascular plexus, endothelial cells tend to be quiescent (12). As the tumor invades peripheral tissues, it gradually develops heavy vascularization, disordered vascular structure, and endothelial anergy, which can result in less immune infiltration, drug delivery, and aggravated hypoxia (13, 14). Persistent hypoxia and an immunosuppressive state will deteriorate the tumor microenvironment (TME), which promotes tumor metastasis and drug resistance (15, 16). Therefore, angiogenesis is considered a key physiopathological process of tumorigenesis. Antiangiogenic treatment is used to inhibit angiogenesis so that the proliferation of the tumor would be suppressed. In recent years, angiogenesis inhibitors are widely used in the targeted therapy of osteosarcoma patients. While they show good efficacy, they cannot significantly improve the prognosis of patients and even lead to drug resistance. It suggests that angiogenesis is closely related to the occurrence and development of osteosarcoma, but the understanding of its mechanism is still insufficient. Recent evidence indicated that antiangiogenic treatment can also block the transport of immune cells and drugs, leading to a paradoxical increase in metastasis (17). Tumor vessel normalization was deemed as a more reliable treatment. Tumor vessel normalization can significantly improve blood perfusion and reduce vascular permeability (18, 19). The increased density of functional vessels can prevent heterogeneity in blood flow within the tumor region (20). Additionally, the crosstalk between vessels and immune cells was important for the effect of immunotherapy. It is well-known that adhering to the vascular surface is the first step for immune cells to attack tumor cells. Tumor-related endothelial cells express low levels of cell adhesion molecules and lead to endothelial anergy, preventing the trafficking of immune cells (21, 22). Moreover, functional vessels can increase the infiltration of immune cells, contributing to a better outcome in immunotherapy (23). On the other hand, the latest study illustrated that immunotherapy may promote tumor vessel normalization, and increased perfusion of tumor vessels was expected to be a predictive biomarker of response to immunotherapy (24, 25). All pieces of evidence indicated that evaluation of the vessel state may help to estimate the immune infiltration condition and further identify the potential patient who is suitable for immunotherapy. Thus, we used “vessel state,” defined by expressions of adhesion molecules and vessel normalization-related genes, to evaluate the function of vessels in tumors.

The TME is essential for tumorigenesis, invasion, and immune infiltration, which makes it a prospective target to predict patients’ prognosis and therapy efficacy (26, 27). Stromal and immune infiltration in the TME were considered important elements for immunotherapy (28). Stromal cells, including cancer-associated fibroblasts (CAFs), pericytes, and mesenchymal stem cells, can prevent immune infiltration (29, 30). A high expression of checkpoint molecules and a large amount of both immune cells and stromal cells were considered immune resistance state, which may be appropriate for ICI treatment in OS (31, 32). Evaluation of the TME can provide evidence for immunotherapy in OS patients.

In our work, 93 OS patients were divided into two clusters according to the expression of angiogenesis-related genes (ARGs). We compared the vessel state and the TME in the two subgroups and identified prognostic hub gens between them. ARG_score showed a correlation with angiogenesis and characterized the immune landscape of OS. Finally, we constructed a risk score model and a prediction model of OS which were proved to be reliable.

## Materials and methods

### Data collection

The bulk RNA-seq data (count data) of OS patients were obtained from the Therapeutically Applicable Research to Generate Effective Treatments (TARGET) database (https://ocg.cancer.gov/programs/target/). Samples lacking clinical information were excluded and 93 of 101 patients with matched clinical information were finally enrolled in our study (**Supplementary Table 1**). “DESeq2” R package was performed to normalize the gene expression dataset and made variance stabilizing transformation (VST) for downstream analysis (**Supplementary Table 2**). GSE21257 (19 primary OS samples and 34 metastatic OS samples) was downloaded from the Gene Expression Omnibus (GEO) database (https://www.ncbi.nlm.nih.gov/geo/) and used as a validation dataset. All of the sample information from the GEO database can be seen in **Supplementary Table 3**. ARGs were obtained from HALLMATK_ANGIOGENESIS and a previous study (33, 34) (**Supplementary Table 4**) (https://www.gsea-msigdb.org/gsea/msigdb/).

### Unsupervised clustering analysis of ARGs

After removing undetected genes (*CCN2, IGF*), 41 ARGs were identified in the TARGET dataset. The “ConsensusClusterPlus” R package was used to classify patients in the TARGET dataset with an unsupervised clustering method. The optimal value of *k* was selected according to the following criteria: a) a higher intragroup correlation and a lower intergroup correlation; b) the cumulative distribution function (CDF) curve increased smoothly, while the Delta area increased gradually; and c) no subgroups have a small sample size. Based on the criteria, *k* = 2 was chosen as the appropriate number of clusters. The survival curve was calculated by the “survival” R package. “pheatmap” R package was used to visualize the expression of ARGs in the two angiogenesis subgroups.

### Vessel state and TME analysis of angiogenesis subgroups

To evaluate the vessel state of the two subgroups, the expressions of adhesion molecules and vessel normalization-related genes were compared. To explore the TME between the angiogenesis subgroups, two independent algorithms were performed to estimate two main components in the TME: a) 64 kinds of tumor-related cells were calculated by xCell, which used an enrichment score to represent cell fraction (35). We extracted the enrichment score of 23 stromal cells and compared their scores between the two subgroups. “xCell” R package was used to complete the analysis. b) Immune cell fraction and immune process were analyzed by single-sample gene set enrichment analysis (ssGSEA), which evaluates 29 immune cells and immune processes using a gene signature-based method (36, 37). The “GSVA” and “GSEABase” R packages were applied.

### Identification of differentially expressed genes in the angiogenesis subgroups and function enrichment analysis

“DESeq2” R package was performed to identify differentially expressed genes (DEGs) between the two angiogenesis subgroups. |Fold change (FC)| >2 and adjusted *p*-value<0.05 were used as the filter criteria. All DEGs were divided into the upregulated and downregulated groups based on their FC for further analysis. Gene Ontology (GO) and Kyoto Encyclopedia of Genes and Genomes (KEGG) were performed by the “clusterProfiler” R package using a *q* value<0.05 as statistically significant enrichment. GSEA 4.2.3 was used for gene set enrichment analysis. Hallmark gene sets were obtained from the MSigDB. A *p*-value<0.05 and |normalized enrichment score (NES) | >1.5 were considered as significant differences between the two subgroups.

### Protein–protein interaction and hub gene analysis

To construct the protein–protein interaction (PPI) networks of the upregulated and downregulated groups, the online Search Tool for the Retrieval of Interacting Genes (STRING) database was used, and the minimum required interaction score was 0.4. Molecular Complex Detection (MCODE) of Cytoscape 3.9.1 was applied to identify the hub genes in the PPI networks of upregulated and downregulated groups. Parameters were set as follows: degree cutoff was 2 and node score cutoff was 2.

### Construction and validation of the risk score model based on the ARG_score

To identify the prognostic significance of DEGs, hub genes in the upregulated and downregulated groups were analyzed by the univariate Cox regression. Genes with a *p*-value<0.05 have been selected. To avoid overfitting, the least absolute shrinkage and selection operator (LASSO) regression was used with the “glmnet” R package. The remaining genes were then analyzed by the multivariate Cox stepwise regression using the AIC method. The formula of ARG_score was as follows:


ARG_score=∑(i) Coefficient gene(i)∗Expression gene(i)

Coefficient means the *β* value of multivariate Cox stepwise regression, Expression means the normalized gene expression (log-transformed), and gene(*i*) means prognostic hub genes in the risk score model. Patients were divided into high- and low-risk scores based on the median of their ARG_score. The Kaplan–Meier (K-M) survival analysis and time-dependent receiver operating characteristic (ROC) curve were conducted by the “survival” and “timeROC” R packages to evaluate the prognostic significance and discrimination of the risk score model built by prognostic hub genes. 3D principal component analysis (PCA) was calculated by the “plotly” R package. GSE21257 was used as a validation dataset to verify the reliability of the risk score model.

### Angiogenesis and immune analysis of the ARG_score

To explore the relationship between ARGs and ARG_score, correlation analysis was performed between the expression of ARGs and ARG_score in TARGET and GSE21257. The interactions of ARGs and prognostic hub genes were visualized by the “GENEMANIA” plugin of Cytoscape. To analyze the relationship between immune cells and ARG_score, correlation analysis was applied. Furthermore, the “estimate” R package was utilized to calculate the stromal score and immune score in the low- and high-risk groups, which were classified by ARG_score. Finally, the expression of checkpoint molecules was also compared in the low- and high-risk groups.

### Prediction of response to immunotherapy and drug sensitivity

To evaluate whether ARG_score can predict the response to immunotherapy, two independent approaches were used: a) tumor inflammation signature (TIS), which was applied to predict the response to anti-PD-1 therapy (38), and b) Immune Cell Abundance Identifier (ImmuCellAI), another tool to predict the response to immune checkpoint blockade therapy (39).A higher TIS score and ImmuCellAI score represented better immunotherapy response. To evaluate the differences in therapeutic efficacy in targeted treatment between the low- and high-risk groups, the “oncoPredict” R package was applied. Drug sensitivity information was obtained from the Genomics of Drug Sensitivity in Cancer (GDSC) database (https://www.cancerrxgene.org/), which has characterized 1,000 human cancer cell lines and screened them with hundreds of compounds.

### Establishment and validation of the predictive nomogram

To construct a more efficient prediction model, clinical features (age, gender, condition of metastasis) and ARG_score of the TARGET dataset were analyzed by univariate Cox regression and multivariate Cox regression. Independent prognostic indicators were identified with *p*-value<0.05 in both univariate and multivariate Cox regressions. An interactive nomogram was performed by the “regplot” R package to predict the 1-, 3-, and 5-year overall survival. A calibration curve was drawn to test the accuracy of prediction of the 1-, 3-, and 5-year overall survival. K-M survival analysis and time-dependent ROC curve were also performed to evaluate the significance and discrimination of the 1-, 3-, and 5-year overall survival. GSE21257 containing clinical features was used as a validation dataset to verify the reliability of the prediction model.

### Specimens and immunohistochemistry

This study was approved by the Ruijin Hospital Ethics Committee, Shanghai Jiao Tong University School of Medicine (IRB protocol number: KY2020-395). Informed consent was obtained from each patient when they were hospitalized. Tissue microarray (TMA) constructed by 110 osteosarcoma tissues (96 primary osteosarcoma patients and 14 recurrent osteosarcoma patients) was used as the experimental group, and 6 normal bone tissues were used as the control group. TMA is an effective high-throughput technique platform for the study of tumor molecular pathology. All the tumor samples were histologically confirmed as osteosarcoma and reviewed independently by two pathologists at Ruijin Hospital. The information of all patients is shown in **Supplementary Table 5**. The process of constructing the TMA was similar to that of a previous report (40). In brief, a hole was generated in an empty paraffin block which was prepared at first. Then, a cylindrical tissue sample was removed from the paraffin block which has been confirmed as osteosarcoma. Finally, the cylindrical tissue sample was placed in the premade hole in the empty paraffin block.

Immunohistochemical staining of GALNT14 and VCAM1 was performed as follows: specimens were fixed with 10% formalin and embedded in paraffin. Then, the paraffin-embedded tissues were cut into slices of 4 μm thickness. After heat-induced antigen retrieval, anti-GALNT14 (Proteintech, Wuhan, Chian, 16939-1-AP, 1:200) and anti-VCAM1 (Abcam, MA, USA, ab134047,1:250) were used to incubate sections at 4°C overnight. Slices were covered with horseradish peroxidase-coupled goat anti-rabbit secondary antibody (CST, 7074, MA, USA, 1:200) at room temperature for 1 h and stained with diaminobenzidine.

By multiplying the intensity of staining (0, no staining; 1, yellow; 2, pale brown; 3, dark brown) with a positive cell rate (0,<5%; 1, 5%–25%; 2, 26%–50%; 3, 51%–75%; 4, 76%–100%), we obtained the IHC score to represent the expression of GALNT14 and VCAM1.

### Quantitative real-time PCR

To verify the expression of prognostic hub genes, the total RNAs of three human osteosarcoma cell lines (MING/HOS, 143B, Well5) and one human osteoblast cell line (hFOB 1.19) were isolated by RNAsimple Total RNA Kit (TIANGEN, China). cDNA was obtained with HyperScript III 1st Strand cDNA Synthesis Kit (NovaBio, China) after removing gDNA. Finally, quantitative real-time PCR (qRT-PCR) was performed by LightCycler480 II, and GAPDH was used as an internal control gene. Validation of each gene was performed three times as biological replications. The primer sequences of the prognostic hub genes are listed below:

GALNT14: CAGGACCATCCGCAGTGTATTA (sense primer)
GALNT14: ACCAGACCTTGCCGTTCATTAT (antisense primer)
MUC1: TATCTCATTGCCTTGGCTGTCT (sense primer)
MUC1: TGCTGGGTTTGTGTAAGAGAGG (antisense primer)
GADPH: GGAAGCTTGTCATCAATGGAAATC (sense primer)
GADPH: TGATGACCCTTTTGGCTCCC (antisense primer)

### Statistical analysis and visualization

R software (version 4.1.2) and Perl (version 5.16.2) were conducted to process, analyze, and visualize data. The hazard ratio (HR) was calculated by univariate and multivariate Cox regressions. Student’s *t*-test was used for the comparison of variables that obey normal distribution, while the Wilcoxon rank-sum test was used for non-normally distributed variables. The Shapiro–Wilk test was applied for the normality test. A two-sided *p*-value<0.05 was deemed as statistically significant. “ggpubr” and “ggplot2” R packages were applied for visualization.

## Results

### Identification of the angiogenesis subgroups in OS

**Figure 1:**
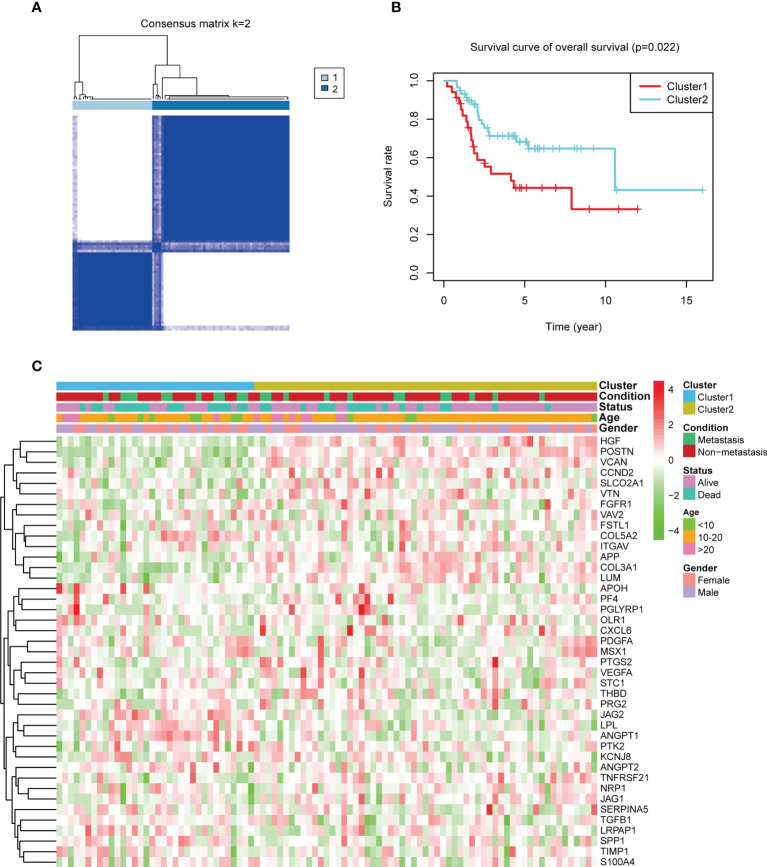
Consensus clustering by angiogenesis-related genes (ARGs) and their expression in the two angiogenesis subgroups. **(A)** The consensus matrix heatmap showed that *k* = 2 can divide samples into two subgroups well. **(B)** Kaplan–Meier plots comparing the overall survival between the two subgroups (log-rank *p-*value = 0.022). **(C)** A heatmap about the expression of ARGs and clinical features in the two subgroups. Green means low expression and red means high expression.

The analysis process of our study is shown in **Supplementary Figure 1**. To illustrate the effect of ARGs in OS, patients in TARGET were selected to perform consensus clustering. According to the consensus matrix heatmaps, CDF curve, and Delta area curve, we divided patients into two angiogenesis subgroups (**Figure 1A**, **Supplementary Figures 2A–I**). There were 34 patients in cluster 1 and 59 patients in cluster 2. K-M survival curve indicated that cluster 2 had a better overall survival (log-rank *p*-value = 0.022, **Figure 1B**) and event-free survival (log-rank *p*-value = 0.017, **Supplementary Figure 3A**) than cluster 1. The PPI network presented the interactions of ARGs, indicating the potential co-expression between ARGs (**Supplementary Figure 3B**). The heatmap showed the gene expression of ARGs between the two angiogenesis subgroups with their matched clinicopathological characteristics (**Figure 1C**). To investigate the specific ARGs differentially expressed in the two subgroups, a boxplot was performed and we found that 6 ARGs (*SPP1*, *LPL*, *JAG2*, *ANGPT1*, *ANGPT2*, and *APOH*) were upregulated in cluster 1 while 13 ARGs (*COL3A1*, *LUM*, *APP*, *POSTN*, *VCAN*, *JAG1*, *TNFRSF21*, *CCND2*, *HGF*, *SLCO2A1*, *PTGS2*, *VTN*, and *CXCL6*) were upregulated in cluster 2 (**Supplementary Figure 3C**). It seemed that different ARGs played different roles in prognosis.

### Vessel state analysis of the angiogenesis subgroups

**Figure 2:**
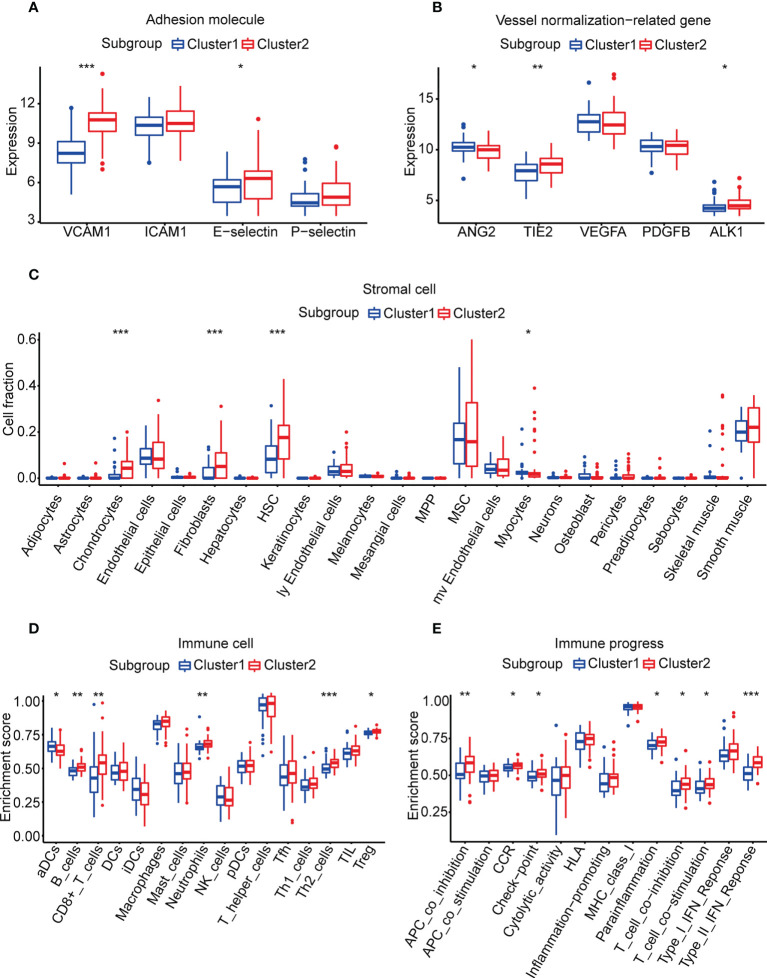
Vessel state and TME in the two subgroups. **(A)** Expression of adhesion molecules. **(B)** Expression of vessel normalization-related genes. **(C)** Enrichment scores of stromal cells calculated by xCell. **(D, E)** Enrichment scores of 29 immune cells and immune processes calculated by ssGSEA analysis. (*p< 0.05, **p< 0.01, ***p< 0.001).

We compared four major adhesion molecules between the angiogenesis subgroups, and *VCAM1* and E-selectin were significantly highly expressed in cluster 2. The median expression of *ICAM1* and P-selectin in cluster 1 was also lower than that in cluster 2 (**Figure 2A**). Vessel normalization-related genes were also associated with vessel state. ANG2/TIE2, VEGFA/PDGFB, and BMP9/ALK1 signaling were important in angiogenesis and vessel normalization (41, 42), and the downregulation of ANG2 and VEGFA and the upregulation of others contributed to vascular normalization (43, 44). We assessed the expression of *ANG2, TIE2, VEGFA, PDGFB*, and *ALK1* in the two subgroups (*BMP9* failed to be detected in the gene expression matrix). It revealed that *TIE2* and *ALK1* were remarkably higher in cluster 2, while *ANG2* was obviously higher in cluster 1. The median expression of *PDGFB* was also higher in cluster 2, and the opposite result can be seen in *VEGFA* (**Figure 2B**). The results above indicated that cluster 2 may have a better vessel state, which may result in a better immune infiltration and prognosis.

### TME analysis of the angiogenesis subgroups

Due to the differences of vessel state in the two subgroups, we speculated that there were also differences in the TME between the two subgroups. The amounts of chondrocytes, fibroblasts, HSCs, and myocytes were significantly different in the two subgroups according to the results of xCell (**Figure 2C**, **Supplementary Table 6**). However, there was no statistical difference between the abundance of these stromal cells and overall survival according to the result of univariate Cox regression (*p*-value > 0.05, **Supplementary Table 7**).

For immune cells and immune processes, ssGSEA was used to calculate their enrichment scores. It showed that five immune cells (B cells, CD8^+^ T cells, neutrophils, Th2 cells, and Tregs) and seven immune functions (APC co-inhibition, CCR, checkpoint, parainflammation, T-cell co-inhibition, T-cell co-stimulation, and type II IFN response) were highly enriched in cluster 2 (**Figures 2D, E**, **Supplementary Table 8**). To identify factors related to prognosis, univariate Cox regression was applied and CD8^+^ T cells, T-cell co-inhibition, checkpoint, APC co-inhibition, and Th2 cells were considered to be significantly associated (*p*-value< 0.05, **Supplementary Table 7**). Then, we divided each factor into a high score and a low score according to the median of enrichment scores. The K-M survival curve illustrated that only CD8^+^ T cells and T-cell co-inhibition were statistically related to overall survival (**Supplementary Figures 3D, E**). CD8^+^ T cells had been reported to induce immunosensitivity and T-cell co-inhibition meant the sensitivity of ICI. These results suggested that the better survival of cluster 2 may benefit from the higher infiltration levels of immune cells.

### Identification of DEGs, function enrichment analysis, and hub gene discovery

**Figure 3:**
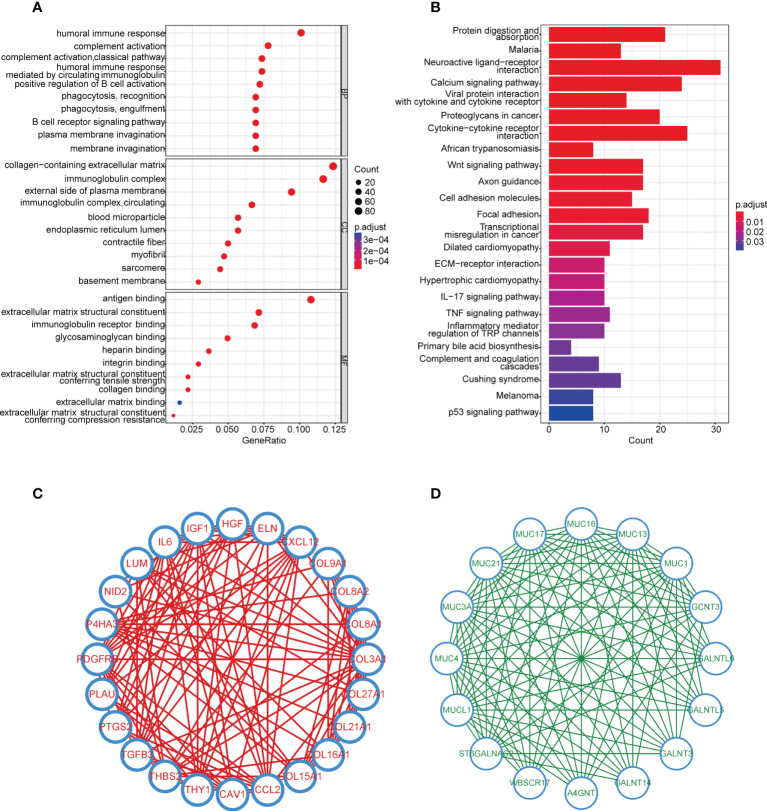
Enrichment analysis and hub genes of DEGs in the two subgroups. **(A)** GO enrichment analysis of upregulated genes in cluster 2, cutoff: *q* value<0.05. **(B)** KEGG pathway analysis of upregulated genes in cluster 2, cutoff: *q* value<0.05. **(C, D)** Hub genes in upregulated and downregulated genes analyzed by MCODE.

To further explore the difference between cluster 1 and cluster 2, 4,376 DEGs between the two subgroups were found (**Supplementary Table 9**). DEGs upregulated and downregulated in cluster 2 were 960 and 3,416, respectively. GO analysis indicated that DEGs highly expressed in cluster 2 were mainly enriched in immune-related processes (complement activation, phagocytosis and recognition, humoral immune response, and so on) (**Figure 3A**, **Supplementary Table 10**). Furthermore, the results of the KEGG analysis illustrated that more immune and cell adhesion-related signaling pathways were enriched in cluster 2 (such as cytokine receptor interaction, cell adhesion molecules, focal adhesion, and so on), suggesting that cluster 2 represented prominent immunocompetence (**Figure 3B**, **Supplementary Table 11**). Moreover, the GSEA results showed that 12 biological processes were significantly upregulated in cluster 2, including interferon-gamma response, KRAS signaling-up, xenobiotic metabolism, and so on (**Supplementary Figures 4A–L**, **Supplementary Table 12**).

In order to discover the hub genes, MCODE was applied. Twenty-four and 16 hub genes were found in upregulated and downregulated DEGs, respectively (**Figures 3C, D**). These 40 hub genes would be studied next.

### Construction and validation of the angiogenesis-related risk score model

**Figure 4:**
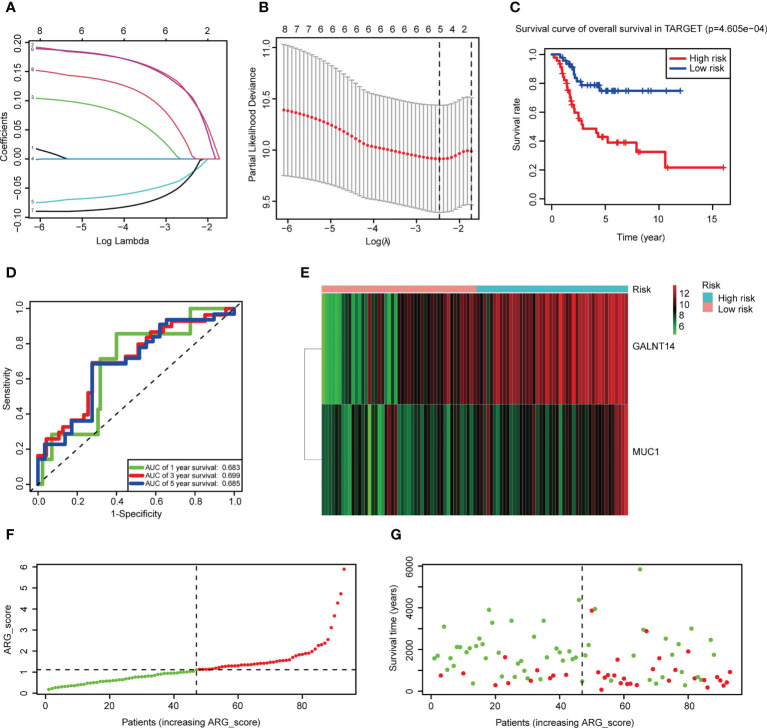
Construction of the risk score model. **(A)** LASSO regression was used to screen the prognostic genes. **(B)** Cross-validation indicated that five genes can be used in multivariate Cox regression. **(C)** K-M plots comparing overall survival between the low- and high-risk groups in the Therapeutically Applicable Research to Generate Effective Treatments (TARGET) dataset (log-rank *p*-value = 4.605e−4). **(D)** Time-dependent ROC curves to predict the discrimination of the 1-, 3-, and 5-year survival according to the risk score model in the TARGET dataset. **(E)** A heatmap of two prognostic hub genes of the TARGET dataset in the two subgroups. Red means high expression and green means low expression. **(F, G)** Ranked dot and scatter plots of the TARGET dataset showing the ARG_score distribution and patient survival status.

After selecting the hub genes from DEGs, we investigated whether these genes were associated with survival. Univariate Cox regression was performed and eight hub genes remained with a *p*-value<0.05 (**Supplementary Table 13**). To avoid overfitting, LASSO regression was used and five genes were independent of each other (**Figures 4A, B**). Finally, we applied multivariate Cox stepwise regression, leaving two genes (*GALNT14* and *MUC1*) to construct the risk score model (**Supplementary Table 13**). ARG_score was calculated as follows: (0.2624 * expression of *GALNT14*) + (0.2547 * expression of *MUC1*). Patients were distinguished as low and high risk based on the median ARG_score. It revealed that the low-risk group had a significantly longer overall survival (**Figure 4C**, log-rank *p*-value = 4.605e−4). The time-dependent ROC curves showed that the area under the curve (AUC) values of the 1-, 3-, and 5-year overall survival were 0.683, 0.699, and 0.685, respectively (**Figure 4D**).

*GALNT14* and *MUC1* had been reported as oncogenes in tumors, and both were overexpressed in the high-risk group (**Figure 4E**). PCA analysis was performed and patients can be divided into two groups according to the risk score model **(Supplementary Figure 5A**). Ranking patients by ARG_score from smallest to largest, the one who had a higher score tended to have a shorter survival time (**Figures 4F, G**). External validation demonstrated that the risk score model can also classify patients of GSE21257 into low and high risk, and significantly worse overall survival was also observed in the high-risk group (**Supplementary Figure 5B**, log-rank *p*-value = 8.253e−3). The AUC values of 1-, 3-, and 5-year overall survival were even better in GSE21257 (0.781, 0.749, and 0.817, respectively, **Supplementary Figure 5C**). The same results of the relationship between ARGs and overall survival can also be seen in GSE21257 (**Supplementary Figures 5D, F**). These results indicated that ARG_score can well predict the prognosis of OS patients, and a lower ARG_score was also observed in cluster 2 which had a better overall survival (**Supplementary Figure 6A**).

### Association of ARG_score with angiogenesis and immune infiltration

**Figure 5:**
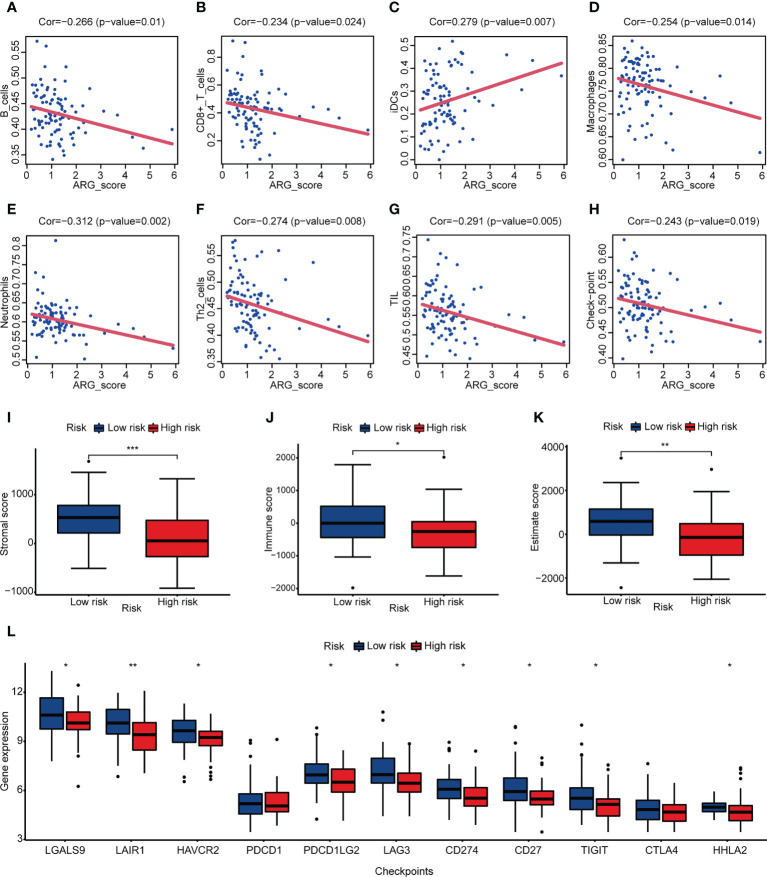
Correlation between ARG_score and immune infiltration. **(A–H)** Correlation analysis between ARG_score and immune cell/checkpoint. **(I–K)** Stromal score, immune score, and estimate score of the low- and high-risk groups. **(L)** Expression levels of checkpoint molecules in the low- and high-risk groups. (*p< 0.05, **p< 0.01, ***p< 0.001).

To investigate the relationship between ARG_score and angiogenesis, PPI analysis was applied and showed the interactions between prognostic hub genes and ARGs (**Supplementary Figure 6B**). We also found that both ARG_scores in TARGET and GSE21257 were significantly correlated with most of the ARGs which were differentially expressed in the two angiogenesis subgroups (**Supplementary Figure 6C**). We then evaluated the association of ARG_score and immune infiltration. Correlation analysis illustrated that a lower ARG_score was related to a higher fraction of immune cells except for iDCs (**Figures 5A–H**). A higher immune score and stromal score suggested that the low-risk group may represent an immune-resistant state (**Figures 5I, J**). A higher estimate score in the low-risk group was also closely associated with a lower purity of tumor cells (**Figure 5K**). A previous study considered that OS patients who had an immune “hot” signature tended to have an adaptive immune resistance state (45). We further compared the expression of checkpoint molecules in the low- and high-risk groups, and most of them were significantly overexpressed in the low-risk group, suggesting that a lower ARG_score may potentially benefit from ICI (**Figure 5L**).

### The value of ARG_score in the prediction of response to immunotherapy and drug sensitivity

**Figure 6:**
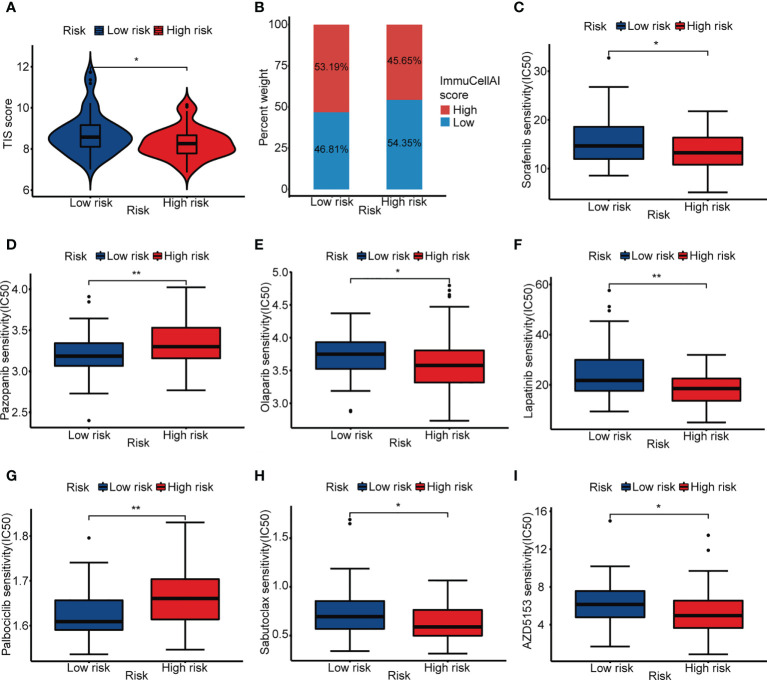
Immunotherapy response prediction and drug sensitivity. **(A)** ITS score of the low- and high-risk groups. **(B)** Proportion of high and low ImmuCellAI score in the low- and high-risk groups. **(C–I)** IC_50_ of sorafenib, pazopanib, olaparib, lapatinib, palbociclib, sabutoclax, and AZD5153 in the low- and high-risk groups. (*p< 0.05, **p< 0.01).

To further explore the prediction ability of ARG_score for clinical response to immunotherapy, we compared the TIS score between the low- and high-risk groups. It showed a higher TIS score in the low-risk group, indicating that ICI may be effective in patients with a lower ARG_score (**Figure 6A**, **Supplementary Table 14**). Moreover, the low-risk group had a higher proportion of patients with a high ImmuCellAI score, which represented a better immunotherapy response (**Figure 6B**, **Supplementary Table 15**). Recent studies prompted that immunotherapy combined with targeted therapy may achieve better efficacy. We assessed some targeted drugs that may potentially benefit OS patients, including the angiogenesis inhibitors (sorafenib and pazopanib), PARP inhibitor (olaparib), HER2 inhibitor (lapatinib), CDK4/6 inhibitor (palbociclib), Bcl-2 inhibitor (sabutoclax), and mTOR inhibitor (AZD5153). It indicated that the low-risk group was more sensitive to pazopanib and Palbociclib, while the high-risk group was more appropriate to be treated with sorafenib, olaparib, lapatinib, sabutoclax, and AZD5153 (**Figures 6C–I**, **Supplementary Table 16**). These results showed that ARG_score was also related to drug sensitivity.

### Development and validation of the prediction model in OS

**Figure 7:**
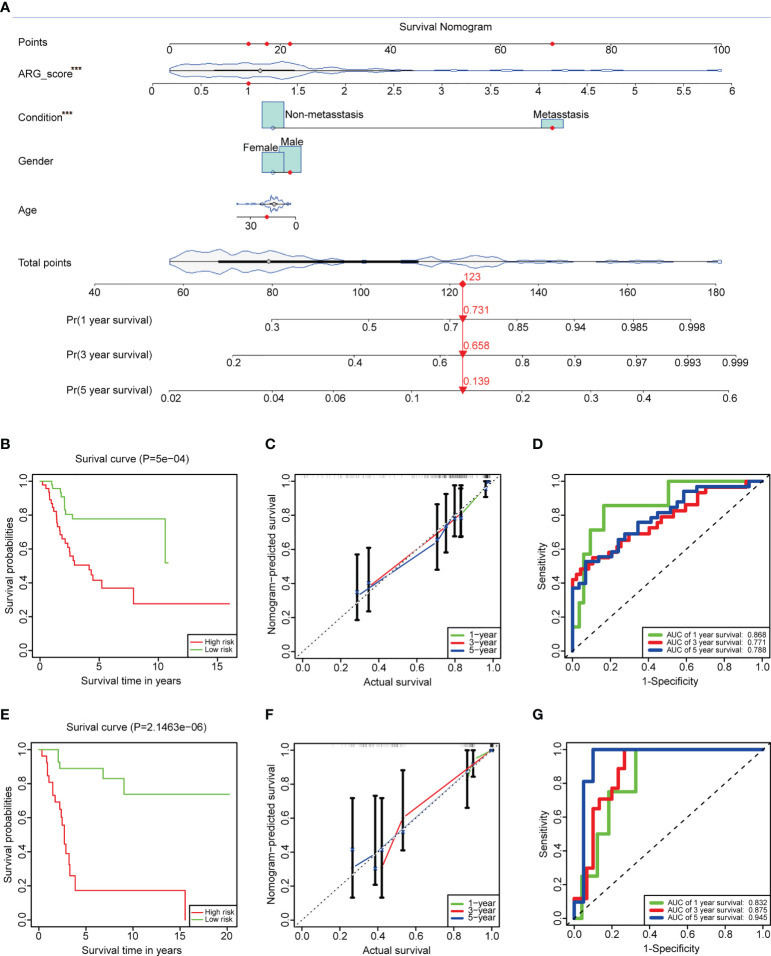
Construction and validation of the prediction model. **(A)** An interactive nomogram built by ARG_score and clinical features. **(B)** K-M plots comparing the overall survival between the low- and high-risk groups in the TARGET dataset (log-rank *p*-value = 5e−4). **(C)** Calibration curves to analyze the accuracy of the 1-, 3-, and 5-year survival according to the prediction model of the TARGET dataset. **(D)** Time-dependent ROC curves to predict the discrimination of the 1-, 3-, and 5-year survival according to the prediction model of the TARGET dataset. **(E–G)** K-M plots, calibration curves, and time-dependent ROC curves in GSE21257, which served as a validation dataset. The symbol *** represents that ARG_score and condition were identified as independent factors in the prediction model, which had a p value < 0.001.

Clinical features always affect the prognosis of patients. To make our prediction model more effective, ARG_score and clinical information (age, gender, and condition) of the TARGET dataset were used to build an interactive nomogram, which predicted the 1-, 3-, and 5-year overall survival (**Figure 7A**). In accordance with the prediction model, patients can be divided into low- and high-risk groups, too. The low-risk group still represented a good outcome (**Figure 7B**, log-rank *p*-value = 5e−4). In order to access the accuracy and discrimination of the prediction model, calibration curves and time-dependent ROC curves were performed. The AUC values of the 1-, 3-, and 5-year overall survival were 0.868, 0.771, and 0.788, respectively (**Figures 7C, D**). In the validation dataset, the K-M curve showed a more significant benefit in overall survival in the low-risk group (**Figure 7E**, log-rank *p*-value = 2.146e−6). The calibration curves and ROC curves demonstrated the reliability of the prediction model (the AUC values of the 1-, 3-, and 5-year overall survival were 0.832, 0.875, and 0.945, respectively; **Figures 7F, G**). Additionally, ARG_score and condition were identified as independent factors in the prediction model, which had a *p*-value<0.05 in both univariate and multivariate Cox regression analyses (**Supplementary Figures 6D, E**, **Supplementary Table 13**).

### Immunohistochemistry and qRT-PCR

**Figure 8:**
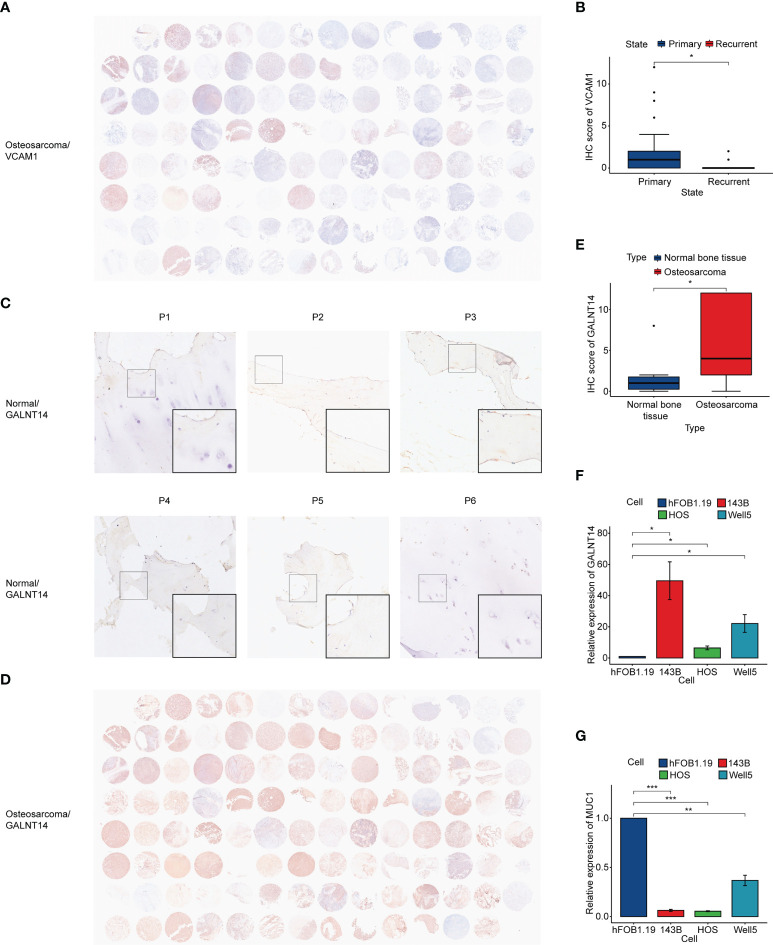
Immunohistochemistry and qRT-PCR. **(A)** Immunohistochemistry of VCAM1 in osteosarcoma tissue. **(B)** IHC score of VCAM1 between the primary osteosarcoma group and the recurrent osteosarcoma group. **(C)** Immunohistochemistry of GALNT14 in normal bone tissue. **(D)** Immunohistochemistry of GALNT14 in osteosarcoma tissue. **(E)** IHC score of *GALNT14* between normal bone tissue and osteosarcoma tissue. **(F, G)** qRT-PCR results of *GALNT14* and *MUC1* in osteoblast cell line and three osteosarcoma cell lines. (*p< 0.05, **p< 0.01, ***p< 0.001).

To verify the relationship between vessel state and prognosis, immunohistochemical staining was performed (**Figure 8A**). The expression of VCAM1, a classical adhesion molecule, was used to evaluate the vessel state of the osteosarcoma tissue. Results showed that the expression of VCAM1 in primary osteosarcoma patients was significantly higher than that in recurrent osteosarcoma patients (**Figure 8B**), which suggested that a better vessel state may be related to a better outcome.

In addition, we accessed the expression of prognostic hub genes. It was observed that GALNT14 was significantly highly expressed in osteosarcoma (**Figures 8C–E**). qRT-PCR showed that the expression of *GALNT14* in osteosarcoma cell lines was also higher than that in the osteoblast cell line, while the opposite result can be seen in *MUC1* (**Figures 8F, G**).

## Discussion

Recently, many studies have paid attention to the process of angiogenesis in tumors. However, most of them emphasized on antiangiogenic therapy and tumor vessel normalization. The roles of vessel state and immune infiltration affected by angiogenesis have not been fully explored, especially in OS. It was shown in the present study that 41 angiogenesis-related genes can well divide patients into two angiogenesis subgroups. Longer overall survival, better vessel state, and more immune infiltration were observed in cluster 2. DEGs of these two subgroups were identified and ARG_score was calculated by two prognostic hub genes. The accuracy and discrimination of the risk score model indicated that ARG_score performed a robust and effective prediction ability. The correlation of ARG_score and ARGs, immune infiltration, ICI, and drug sensitivity suggested that ARG_score can also estimate the angiogenesis and immunotherapy efficacy in OS patients. The prediction model constructed by ARG_score and clinical features made overall survival prediction more reliable. The results above may assist in a better understanding of the relationship between angiogenesis and immunotherapy in OS.

The selection of patients who may respond to immunotherapy has always been a crucial problem in tumor therapy. Tumor mutation burden, microsatellite instability, and expression levels of checkpoint molecules were considered as the signatures to evaluate immunotherapy efficacy (46, 47). Nevertheless, the mutation pattern of OS was remarkably different from other solid tumors. Numerous studies revealed that a high level of structural variants including somatic structural variations, copy number variations, and chromothripsis showed the genomic complexity of OS (48, 49). Only a minority of OS patients benefited from immunotherapy, suggesting that more mechanisms need to be explored for efficacy prediction. We defined vessel state as the vascular structure and endothelial cell function, which have been reported as an important factor of immune infiltration (50, 51). ICAM1, VCAM1, E-selectin, and P-selectin were known as adhesion molecules and mainly expressed on endothelial cells. Downregulation of these genes enabled tumor cells to escape from immune surveillance (52). In our study, a higher expression of adhesion molecules was associated with better prognosis and immune infiltration, which was consistent with previous reports. Vessel normalization can change the abnormal structure of a vessel into a more “mature” phenotype (23). Overexpression of ANG2 and VEGFR promoted the immaturity of the vessel and suppressed the expression of TIE2 and PDGFB which contributed to a mature phenotype (53, 54). Notably, *ANG2* and *VEGFA* were angiogenesis-related genes in our study, suggesting that angiogenesis was associated with vessel state. With a higher expression of adhesion molecules and normalization-related genes, cluster 2 represented a better vessel state. Combined with its good prognosis and immune infiltration, we speculated that vessel state may be a complementary part of immunotherapy efficacy prediction and more experiments should be conducted.

It was generally suggested that patients with more T-cell infiltration would respond better to immunotherapy (55). Patients in cluster 2 had better immune infiltration, especially CD8^+^ T cells. CD8^+^ T cells were proved to be associated with longer overall survival time in our study and clearly defined as an immunotherapy effectiveness signature (56). Nevertheless, the density of Tregs and Th2 cells, which were considered a poor prognosis in some studies, was also higher in cluster 2 (57, 58). In fact, many types of immune cells were discovered to have different roles in different tumors. Decreased Treg cells correlated to shorter overall survival in breast cancer and hepatocellular carcinoma, while it correlated to longer overall survival in colorectal cancer (59–61). Different outcomes related to Th2 cells were also observed in pancreatic cancer and Hodgkin lymphoma (62, 63). It may reveal that the function of immune cells was associated with their interaction with different kinds of tumors. Notably, larger amounts of stromal cells and a higher expression of checkpoints were detected in cluster 2, which meant that cluster 2 may be an adaptive immune-resistant subgroup and was appropriate for ICI treatment (64). Higher enrichment of B cells was also observed in cluster 2. More and more studies regarded that B cells played an important role in the immune response. Immunoglobulin subclass switch events were observed in numerous cancers (65). Intratumoral tertiary lymphoid structures and cytokine release were related to better prognosis and positive response on immunotherapy (66, 67). Importantly, the latest research illustrated that B cells were the strongest prognostic factor in sarcoma, which emphasized the significance of B cells in sarcoma therapy (68).

To further explore the contribution of angiogenesis subgroups to the prognosis of OS patients, we identified two prognosis-associated hub genes, *GALNT14* and *MUC1*. N-acetyl-galactosaminyltransferases (GALNT14) is a member of the polypeptide N-acetylgalactosaminyltransferase (GALNT) family, which can catalyze mucin-type O-glycosylation of proteins (69). It was reported that *GALNT14* was overexpressed in more than 30% of samples from various human malignant tumors (70). As a carcinoma driver gene, *GALNT14* was proved to participate in the tumorigenesis and progression in ovarian cancer, hepatocellular carcinoma, lung cancers, and so on (71–73). Mucin-1 (*MUC1*), a kind of O-glycosylated transmembrane glycoprotein, was the potential biomarker in breast cancer, lung adenocarcinoma, and colorectal adenocarcinoma (74, 75). In other studies, a high expression of *MUC1* in prostate cancer and multiple myeloma may lead to a malignant phenotype (76, 77). Transcriptome analysis showed that a high expression of *GALNT14* and *MUC1* in osteosarcoma was associated with poor prognosis. However, the results of qRT-PCR showed that the expression of *MUC1* in osteosarcoma cell lines was lower than that in osteoblastic cell lines, which may be related to hypermethylation or co-expression inhibition of other genes. More evidence is required. We used these two hub genes to calculate the ARG_score, which showed its correlation with angiogenesis and immune infiltration in our study. Notably, the expressions of *VCAN*, *LPL*, *TNFRSF21*, and *JAG2* were significantly different in the two subgroups and correlated with ARG_score in both TARGET and GSE21257, and they may serve as the core genes of vessel state. Moreover, a lower ARG_score was associated with a larger immune cell abundance and a higher immune score. The results of immunotherapy prediction were in accord with ARG_score. To some extent, ARG_score can reflect the immunocompetence of OS patients. A higher expression of checkpoint molecules and a better ImmuCellAI score were observed in the lower ARG_score, which meant that low-risk patients may respond to ICI therapy. ICIs have proved their efficacy and safety, ARG_score can help classify patients into low- and high-risk groups, and patients with low ARG_score may be appropriate for ICI treatment and have better overall survival. Furthermore, a lower ARG_score was associated with a higher TIS score, which served as a potential predictive biomarker for PD-1 inhibitor combination therapy. Combination therapy can often improve efficacy, and drug sensitivity analysis found that sorafenib, olaparib, lapatinib, sabutoclax, and AZD5153 were more suitable for patients with a lower ARG_score. This may help in clinical decision-making concerning immunotherapy combined with targeted therapy. We concluded that ARG_score can identify OS patients who benefited from immunotherapy and predict their prognosis, and these results were derived from their differential expression in ARGs. As far as we know, our work is the first to elucidate the relationship among angiogenesis, vessel state, immune infiltration, and prognosis in OS, which may contribute to making immunotherapy decisions in OS patients.

Although our findings seem encouraging, there were some limitations in our research. Firstly, we discovered the different vessel states, immune infiltration, and prognoses in the two angiogenesis subgroups, but we did not prove the causality among them. Based on a previous study, we propose to use vessel state as a novel predictive factor of immunotherapy in OS, and more explorations are needed. Moreover, the spatial distribution of cells in the TME was important for the estimation of immunotherapy efficiency (78). Immune cells infiltrating between tumor cells are more likely to exert an antitumor effect than infiltrating between stromal cells. The specific mechanism by which the prognosis-associated hub genes influence the immune effect has also not been explored. This will be the direction of our research in the future.

## Conclusion

Briefly, our integrated analysis of angiogenesis subgroups revealed the relationship between angiogenesis, vessel state, and immune infiltration, and we proposed to identify the patients with better vessel states for immunotherapy. We also constructed a prediction model with prognostic-related hub genes, and it performed well in overall survival prediction. These findings emphasized the importance of angiogenesis and would provide a new perspective for immunotherapy of OS patients.

## Data availability statement

The original contributions presented in the study are included in the article/**Supplementary Material**. Further inquiries can be directed to the corresponding author. All data sets enrolled in our study can be found in Therapeutically Applicable Research to Generate Effective Treatments (TARGET, https://ocg.cancer.gov/programs/target/) and Gene Expression Omnibus (GEO, https://www.ncbi.nlm.nih.gov/geo/).

## Ethics statement

The studies involving human participants were reviewed and approved by the Ruijin Hospital Ethics Committee, Shanghai Jiao Tong University School of Medicine. Written informed consent to participate in this study was provided by the participants’ legal guardian/next of kin. Written informed consent was obtained from the individual(s), and minor(s)’ legal guardian/next of kin, for the publication of any potentially identifiable images or data included in this article.

## Author contributions

JTW and YS designed our work. JTW, ZJ, and YF collected and processed the datasets from the public database. JTW and JW conducted the experiment. JTW, ZJ, and JL performed the statistical analysis. All authors have approved the final version of the manuscript.

## Acknowledgments

We thank all the participants in the public datasets, including TARGET, GEO, MSigDB, GDSC, and BioRender. Many thanks to all the developers of the R packages we used and the developers of Perl. We thank Jun Wang for providing some materials. We also thank Qiyuan Bao, Yueyue Zhu, Jianing Hu, and Mingfeng Yang as they provided some suggestions for our study.

## Conflict of interest

The authors declare that the research was conducted in the absence of any commercial or financial relationships that could be construed as a potential conflict of interest.

## Publisher’s note

All claims expressed in this article are solely those of the authors and do not necessarily represent those of their affiliated organizations, or those of the publisher, the editors and the reviewers. Any product that may be evaluated in this article, or claim that may be made by its manufacturer, is not guaranteed or endorsed by the publisher.

## References

[1] Chen C Xie L Ren T Huang Y Xu J Guo W. Immunotherapy for osteosarcoma: Fundamental mechanism, rationale, and recent breakthroughs. Cancer Lett (2021) 500:1–10. doi: 10.1016/j.canlet.2020.12.024 33359211

[2] Jin W Zhang T Zhou W He P Sun Y Hu S. Discovery of 2-Amino-3-cyanothiophene derivatives as potent STAT3 inhibitors for the treatment of osteosarcoma growth and metastasis. J Med Chem (2022) 65:6710–28. doi: 10.1021/acs.jmedchem.2c00004 35476936

[3] Klein O Kee D Nagrial A Markman B Underhill C Michael M. Evaluation of combination nivolumab and ipilimumab immunotherapy in patients with advanced biliary tract cancers: Subgroup analysis of a phase 2 nonrandomized clinical trial. JAMA Oncol (2020) 6:1405–9. doi: 10.1001/jamaoncol.2020.2814 PMC7393585 32729929

[4] Rodriguez-Ruiz ME Perez-Gracia J. L Rodriguez I Alfaro C Onate C Perez G. Combined immunotherapy encompassing intratumoral poly-ICLC, dendritic-cell vaccination and radiotherapy in advanced cancer patients. Ann Oncol (2018) 29:1312–9. doi: 10.1093/annonc/mdy089 29554212

[5] Friedman CF Hainsworth JD Kurzrock R Spigel DR Burris HA Sweeney CJ. Atezolizumab treatment of tumors with high tumor mutational burden from MyPathway, a multicenter, open-label, phase IIa multiple basket study. Cancer Discov (2022) 12:654–69. doi: 10.1158/2159-8290.CD-21-0450 PMC9394388 34876409

[6] Meric-Bernstam F Larkin J Tabernero J Bonini C. Enhancing anti-tumour efficacy with immunotherapy combinations. Lancet (2021) 397:1010–22. doi: 10.1016/S0140-6736(20)32598-8 33285141

[7] Sharma P Allison JP. The future of immune checkpoint therapy. Science (2015) 348:56–61. doi: 10.1126/science.aaa8172 25838373

[8] Le Cesne A Marec-Berard P Blay J. Y Gaspar N Bertucci F Penel N. Programmed cell death 1 (PD-1) targeting in patients with advanced osteosarcomas: results from the PEMBROSARC study. Eur J Cancer (2019) 119:151–7. doi: 10.1016/j.ejca.2019.07.018 31442817

[9] Boye K Longhi A Guren T Lorenz S Naess S Pierini M. Pembrolizumab in advanced osteosarcoma: Results of a single-arm, open-label, phase 2 trial. Cancer Immunol Immunother (2021) 70:2617–24. doi: 10.1007/s00262-021-02876-w PMC8360887 33580363

[10] Carmeliet P. Angiogenesis in health and disease. Nat Med (2003) 9:653–60. doi: 10.1038/nm0603-653 12778163

[11] Fiedler U Augustin HG. Angiopoietins: a link between angiogenesis and inflammation. Trends Immunol (2006) 27:552–8. doi: 10.1016/j.it.2006.10.004 17045842

[12] Lugano R Ramachandran M Dimberg A. Tumor angiogenesis: causes, consequences, challenges and opportunities. Cell Mol Life Sci (2020) 77:1745–70. doi: 10.1007/s00018-019-03351-7 PMC7190605 31690961

[13] Martin JD Seano G Jain RK. Normalizing function of tumor vessels: Progress, opportunities, and challenges. Annu Rev Physiol (2019) 81:505–34. doi: 10.1146/annurev-physiol-020518-114700 PMC6571025 30742782

[14] Carmeliet P Jain RK. Molecular mechanisms and clinical applications of angiogenesis. Nature (2011) 473:298–307. doi: 10.1038/nature10144 21593862 PMC4049445

[15] Liu Y Cao X. Characteristics and significance of the pre-metastatic niche. Cancer Cell (2016) 30:668–81. doi: 10.1016/j.ccell.2016.09.011 27846389

[16] Sharma A Arambula JF Koo S Kumar R Singh H Sessler JL. Hypoxia-targeted drug delivery. Chem Soc Rev (2019) 48:771–813. doi: 10.1039/c8cs00304a 30575832 PMC6361706

[17] Paez-Ribes M Allen E Hudock J Takeda T Okuyama H Vinals F. Antiangiogenic therapy elicits malignant progression of tumors to increased local invasion and distant metastasis. Cancer Cell (2009) 15:220–31. doi: 10.1016/j.ccr.2009.01.027 PMC2874829 19249680

[18] Carmeliet P Jain RK. Principles and mechanisms of vessel normalization for cancer and other angiogenic diseases. Nat Rev Drug Discov (2011) 10:417–27. doi: 10.1038/nrd3455 21629292

[19] Jain RK. Normalization of tumor vasculature: an emerging concept in antiangiogenic therapy. Science (2005) 307:58–62. doi: 10.1126/science.1104819 15637262

[20] Zheng R Li F Li F Gong A. Targeting tumor vascularization: promising strategies for vascular normalization. J Cancer Res Clin Oncol (2021) 147:2489–505. doi: 10.1007/s00432-021-03701-8 PMC11802031 34148156

[21] Huang Y Kim BYS Chan C. K Hahn SM Weissman IL Jiang W. Improving immune-vascular crosstalk for cancer immunotherapy. Nat Rev Immunol (2018) 18:195–203. doi: 10.1038/nri.2017.145 29332937 PMC5922422

[22] Huinen ZR Huijbers EJM van Beijnum JR Nowak-Sliwinska P Griffioen AW. Anti-angiogenic agents - overcoming tumour endothelial cell anergy and improving immunotherapy outcomes. Nat Rev Clin Oncol (2021) 18:527–40. doi: 10.1038/s41571-021-00496-y 33833434

[23] Goel S Duda DG Xu L Munn LL Boucher Y Fukumura D. Normalization of the vasculature for treatment of cancer and other diseases. Physiol Rev (2011) 91:1071–121. doi: 10.1152/physrev.00038.2010 PMC3258432 21742796

[24] Mpekris F Voutouri C Baish J. W Duda D. G Munn LL Stylianopoulos T. Combining microenvironment normalization strategies to improve cancer immunotherapy. Proc Natl Acad Sci USA (2020) 117:3728–37. doi: 10.1073/pnas.1919764117 PMC7035612 32015113

[25] Wong PP Bodrug N Hodivala-Dilke KM. Exploring novel methods for modulating tumor blood vessels in cancer treatment. Curr Biol (2016) 26:R1161–6. doi: 10.1016/j.cub.2016.09.043 27825457

[26] Bejarano L Jordao MJC Joyce JA. Therapeutic targeting of the tumor microenvironment. Cancer Discov (2021) 11:933–59. doi: 10.1158/2159-8290.CD-20-1808 33811125

[27] Goliwas KF Deshane JS Elmets CA Athar M. Moving immune therapy forward targeting TME. Physiol Rev (2021) 101:417–25. doi: 10.1152/physrev.00008.2020 PMC8428923 32790578

[28] Zhang T Jia Y Yu Y Zhang B Xu F Guo H. Targeting the tumor biophysical microenvironment to reduce resistance to immunotherapy. Adv Drug Deliv Rev (2022) 186:114319. doi: 10.1016/j.addr.2022.114319 35545136

[29] Klemm F Joyce JA. Microenvironmental regulation of therapeutic response in cancer. Trends Cell Biol (2015) 25:198–213. doi: 10.1016/j.tcb.2014.11.006 25540894 PMC5424264

[30] Hu M Huang L. Strategies targeting tumor immune and stromal microenvironment and their clinical relevance. Adv Drug Deliv Rev (2022) 183:114137. doi: 10.1016/j.addr.2022.114137 35143893

[31] Chen YP Zhang Y Lv JW Li YQ Wang YQ He QM. Genomic analysis of tumor microenvironment immune types across 14 solid cancer types: Immunotherapeutic implications. Theranostics (2017) 7:3585–94. doi: 10.7150/thno.21471 PMC5596445 28912897

[32] Srinivasan P Wu X Basu M Rossi C Sandler AD. PD-L1 checkpoint inhibition and anti-CTLA-4 whole tumor cell vaccination counter adaptive immune resistance: A mouse neuroblastoma model that mimics human disease. PloS Med (2018) 15:e1002497. doi: 10.1371/journal.pmed.1002497 29377881 PMC5788338

[33] Liberzon A Birger C Thorvaldsdottir H Ghandi M Mesirov JP Tamayo P. The molecular signatures database (MSigDB) hallmark gene set collection. Cell Syst (2015) 1:417–25. doi: 10.1016/j.cels.2015.12.004 PMC4707969 26771021

[34] Galie M Konstantinidou G Peroni D Scambi I Marchini C Lisi V. Mesenchymal stem cells share molecular signature with mesenchymal tumor cells and favor early tumor growth in syngeneic mice. Oncogene (2008) 27:2542–51. doi: 10.1038/sj.onc.1210920 17998939

[35] Aran D Hu Z Butte AJ. xCell: digitally portraying the tissue cellular heterogeneity landscape. Genome Biol (2017) 18:220. doi: 10.1186/s13059-017-1349-1 29141660 PMC5688663

[36] He Y Jiang Z Chen C Wang X. Classification of triple-negative breast cancers based on immunogenomic profiling. J Exp Clin Cancer Res (2018) 37:327. doi: 10.1186/s13046-018-1002-1 30594216 PMC6310928

[37] Hanzelmann S Castelo R Guinney J. GSVA: gene set variation analysis for microarray and RNA-seq data. BMC Bioinf (2013) 14:7. doi: 10.1186/1471-2105-14-7 PMC3618321 23323831

[38] Ayers M Lunceford J Nebozhyn M Murphy E Loboda A Kaufman DR. IFN-gamma-related mRNA profile predicts clinical response to PD-1 blockade. J Clin Invest (2017) 127:2930–40. doi: 10.1172/JCI91190 PMC5531419 28650338

[39] Miao YR Zhang Q Lei Q Luo M Xie GY Wang H. ImmuCellAI: A unique method for comprehensive T-cell subsets abundance prediction and its application in cancer immunotherapy. Adv Sci (Weinh) (2020) 7:1902880. doi: 10.1002/advs.201902880 32274301 PMC7141005

[40] Dancau AM Simon R Mirlacher M Sauter G. Tissue microarrays. Methods Mol Biol (2010) 576:49–60. doi: 10.1007/978-1-59745-545-9_4 19882257

[41] Wu X Giobbie-Hurder A Liao X Connelly C Connolly EM Li J. Angiopoietin-2 as a biomarker and target for immune checkpoint therapy. Cancer Immunol Res (2017) 5:17–28. doi: 10.1158/2326-6066.CIR-16-0206 28003187 PMC5215959

[42] Ayuso-Inigo B Mendez-Garcia L Pericacho M Munoz-Felix JM. The dual effect of the BMP9-ALK1 pathway in blood vessels: An opportunity for cancer therapy improvement? Cancers (Basel) (2021) 13:5412. doi: 10.3390/cancers13215412 34771575 PMC8582496

[43] Teichert M Milde L Holm A Stanicek L Gengenbacher N Savant S. Pericyte-expressed Tie2 controls angiogenesis and vessel maturation. Nat Commun (2017) 8:16106. doi: 10.1038/ncomms16106 28719590 PMC5520106

[44] Viallard C Audiger C Popovic N Akla N Lanthier K Legault-Navarrete I. BMP9 signaling promotes the normalization of tumor blood vessels. Oncogene (2020) 39:2996–3014. doi: 10.1038/s41388-020-1200-0 32042114

[45] Gill J Gorlick R. Advancing therapy for osteosarcoma. Nat Rev Clin Oncol (2021) 18:609–24. doi: 10.1038/s41571-021-00519-8 34131316

[46] Fumet JD Truntzer C Yarchoan M Ghiringhelli F. Tumour mutational burden as a biomarker for immunotherapy: Current data and emerging concepts. Eur J Cancer (2020) 131:40–50. doi: 10.1016/j.ejca.2020.02.038 32278982 PMC9473693

[47] Gelsomino F Barbolini M Spallanzani A Pugliese G Cascinu S. The evolving role of microsatellite instability in colorectal cancer: A review. Cancer Treat Rev (2016) 51:19–26. doi: 10.1016/j.ctrv.2016.10.005 27838401

[48] Kansara M Teng MW Smyth MJ Thomas DM. Translational biology of osteosarcoma. Nat Rev Cancer (2014) 14:722–35. doi: 10.1038/nrc3838 25319867

[49] Gianferante DM Mirabello L Savage SA. Germline and somatic genetics of osteosarcoma - connecting aetiology, biology and therapy. Nat Rev Endocrinol (2017) 13:480–91. doi: 10.1038/nrendo.2017.16 28338660

[50] Missiaen R Mazzone M Bergers G. The reciprocal function and regulation of tumor vessels and immune cells offers new therapeutic opportunities in cancer. Semin Cancer Biol (2018) 52:107–16. doi: 10.1016/j.semcancer.2018.06.002 PMC6548870 29935312

[51] Johansson-Percival A He B Ganss R. Immunomodulation of tumor vessels: It takes two to tango. Trends Immunol (2018) 39:801–14. doi: 10.1016/j.it.2018.08.001 30153971

[52] Wang HT Lee HI Guo JH Chen SH Liao ZK Huang KW. Calreticulin promotes tumor lymphocyte infiltration and enhances the antitumor effects of immunotherapy by up-regulating the endothelial expression of adhesion molecules. Int J Cancer (2012) 130:2892–902. doi: 10.1002/ijc.26339 21805477

[53] Fukumura D Kloepper J Amoozgar Z Duda DG Jain RK. Enhancing cancer immunotherapy using antiangiogenics: opportunities and challenges. Nat Rev Clin Oncol (2018) 15:325–40. doi: 10.1038/nrclinonc.2018.29 PMC5921900 29508855

[54] Liu Z Wang Y Huang Y Kim BYS Shan H Wu D. Tumor vasculatures: A new target for cancer immunotherapy. Trends Pharmacol Sci (2019) 40:613–23. doi: 10.1016/j.tips.2019.07.001 PMC7925217 31331639

[55] Schneider MA Heeb L Beffinger MM Pantelyushin S Linecker M Roth L. Attenuation of peripheral serotonin inhibits tumor growth and enhances immune checkpoint blockade therapy in murine tumor models. Sci Transl Med (2021) 13:eabc8188. doi: 10.1126/scitranslmed.abc8188 34524861

[56] Erdag G Schaefer JT Smolkin ME Deacon DH Shea SM Dengel LT. Immunotype and immunohistologic characteristics of tumor-infiltrating immune cells are associated with clinical outcome in metastatic melanoma. Cancer Res (2012) 72:1070–80. doi: 10.1158/0008-5472.CAN-11-3218 PMC3306813 22266112

[57] Marangoni F Zhakyp A Corsini M Geels SN Carrizosa E Thelen M. Expansion of tumor-associated treg cells upon disruption of a CTLA-4-dependent feedback loop. Cell (2021) 184:3998–4015.e3919. doi: 10.1016/j.cell.2021.05.027 34157302 PMC8664158

[58] Zheng Z Li YN Jia S Zhu M Cao L Tao M. Lung mesenchymal stromal cells influenced by Th2 cytokines mobilize neutrophils and facilitate metastasis by producing complement C3. Nat Commun (2021) 12:6202. doi: 10.1038/s41467-021-26460-z 34707103 PMC8551331

[59] Bates GJ Fox SB Han C Leek RD Garcia JF Harris AL. Quantification of regulatory T cells enables the identification of high-risk breast cancer patients and those at risk of late relapse. J Clin Oncol (2006) 24:5373–80. doi: 10.1200/JCO.2006.05.9584 17135638

[60] Fu J Xu D Liu Z Shi M Zhao P Fu B. Increased regulatory T cells correlate with CD8 T-cell impairment and poor survival in hepatocellular carcinoma patients. Gastroenterology (2007) 132:2328–39. doi: 10.1053/j.gastro.2007.03.102 17570208

[61] Frey DM Droeser RA Viehl CT Zlobec I Lugli A Zingg U. High frequency of tumor-infiltrating FOXP3(+) regulatory T cells predicts improved survival in mismatch repair-proficient colorectal cancer patients. Int J Cancer (2010) 126:2635–43. doi: 10.1002/ijc.24989 19856313

[62] De Monte L Reni M Tassi E Clavenna D Papa I Recalde H. Intratumor T helper type 2 cell infiltrate correlates with cancer-associated fibroblast thymic stromal lymphopoietin production and reduced survival in pancreatic cancer. J Exp Med (2011) 208:469–78. doi: 10.1084/jem.20101876 PMC3058573 21339327

[63] Schreck S Friebel D Buettner M Distel L Grabenbauer G Young LS. Prognostic impact of tumour-infiltrating Th2 and regulatory T cells in classical Hodgkin lymphoma. Hematol Oncol (2009) 27:31–9. doi: 10.1002/hon.878 18924115

[64] Ribas A. Adaptive immune resistance: How cancer protects from immune attack. Cancer Discov (2015) 5:915–9. doi: 10.1158/2159-8290.CD-15-0563 PMC4560619 26272491

[65] Hu X Zhang J Wang J Fu J Li T Zheng X. Landscape of b cell immunity and related immune evasion in human cancers. Nat Genet (2019) 51:560–7. doi: 10.1038/s41588-018-0339-x PMC6773274 30742113

[66] Shi Y. PLAN b for immunotherapy: Promoting and leveraging anti-tumor b cell immunity. J Control Release (2021) 339:156–63. doi: 10.1016/j.jconrel.2021.09.028 34563591

[67] Helmink BA Reddy SM Gao J Zhang S Basar R Thakur R. B cells and tertiary lymphoid structures promote immunotherapy response. Nature (2020) 577:549–55. doi: 10.1038/s41586-019-1922-8 PMC8762581 31942075

[68] Petitprez F de Reynies A Keung EZ Chen TW Sun CM Calderaro J. B cells are associated with survival and immunotherapy response in sarcoma. Nature (2020) 577:556–60. doi: 10.1038/s41586-019-1906-8 31942077

[69] Shan J Liu Y Wang Y Li Y Yu X Wu C. GALNT14 involves the regulation of multidrug resistance in breast cancer cells. Transl Oncol (2018) 11:786–93. doi: 10.1016/j.tranon.2018.04.003 PMC6058006 29702465

[70] Wagner KW Punnoose EA Januario T Lawrence DA Pitti RM Lancaster K. Death-receptor O-glycosylation controls tumor-cell sensitivity to the proapoptotic ligand Apo2L/TRAIL. Nat Med (2007) 13:1070–7. doi: 10.1038/nm1627 17767167

[71] Lin WR Yeh CT. GALNT14: An emerging marker capable of predicting therapeutic outcomes in multiple cancers. Int J Mol Sci (2020) 21:1491. doi: 10.3390/ijms21041491 PMC7073045 32098271

[72] Yang J Li G Zhang K. MiR-125a regulates ovarian cancer proliferation and invasion by repressing GALNT14 expression. BioMed Pharmacother (2016) 80:381–7. doi: 10.1016/j.biopha.2015.12.027 27133078

[73] Kwon OS Lee H Kong HJ Kwon EJ Park JE Lee W. Connectivity map-based drug repositioning of bortezomib to reverse the metastatic effect of GALNT14 in lung cancer. Oncogene (2020) 39:4567–80. doi: 10.1038/s41388-020-1316-2 32388539

[74] Taylor-Papadimitriou J Burchell J Miles DW Dalziel M. MUC1 and cancer. Biochim Biophys Acta (1999) 1455:301–13. doi: 10.1016/s0925-4439(99)00055-1 10571020

[75] Santos-Silva F Fonseca A Caffrey T Carvalho F Mesquita P Reis C. Thomsen-friedenreich antigen expression in gastric carcinomas is associated with MUC1 mucin VNTR polymorphism. Glycobiology (2005) 15:511–7. doi: 10.1093/glycob/cwi027 15604091

[76] Tagde A Rajabi H Bouillez A Alam M Gali R Bailey S. MUC1-c drives MYC in multiple myeloma. Blood (2016) 127:2587–97. doi: 10.1182/blood-2015-07-659151 PMC4882805 26907633

[77] Yasumizu Y Rajabi H Jin C Hata T Pitroda S Long MD. MUC1-c regulates lineage plasticity driving progression to neuroendocrine prostate cancer. Nat Commun (2020) 11:338. doi: 10.1038/s41467-019-14219-6 31953400 PMC6969104

[78] Carstens JL Correa de Sampaio P Yang D Barua S Wang H Rao A. Spatial computation of intratumoral T cells correlates with survival of patients with pancreatic cancer. Nat Commun (2017) 8:15095. doi: 10.1038/ncomms15095 28447602 PMC5414182

