# Supplementary material for: Vessel state and immune infiltration of the angiogenesis subgroup and construction of a prediction model in osteosarcoma
Source: Front Immunol. 2022 Nov 2;13:992266. doi: 10.3389/fimmu.2022.992266 (PMC9666676; doi:10.3389/fimmu.2022.992266)
Supplement: Supplementary file 2 [file DataSheet_2.pdf]

Supplementary Figure

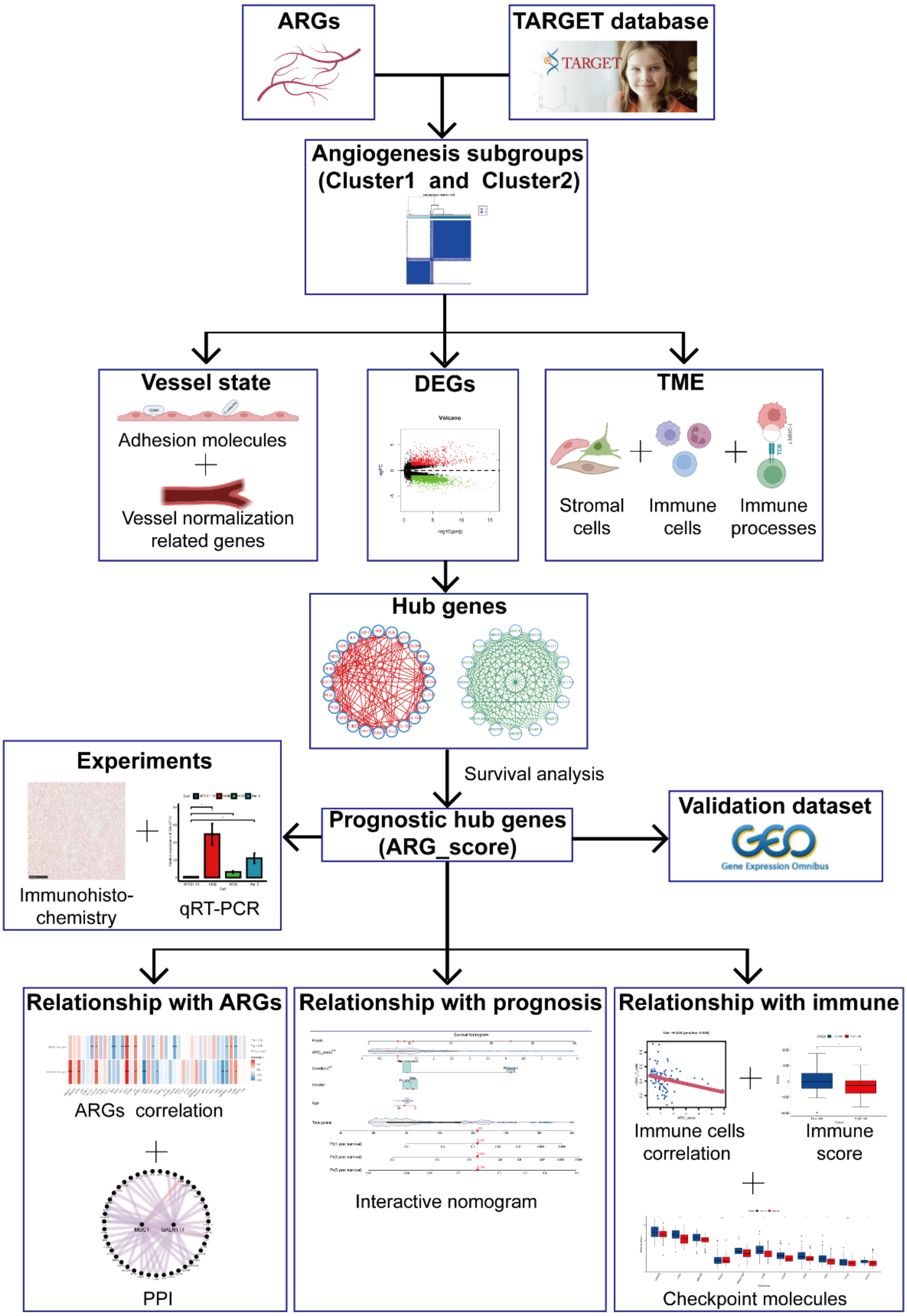

Supplementary Figure 1 Flow chart of our study. (Part of the material was created with BioRender.com)

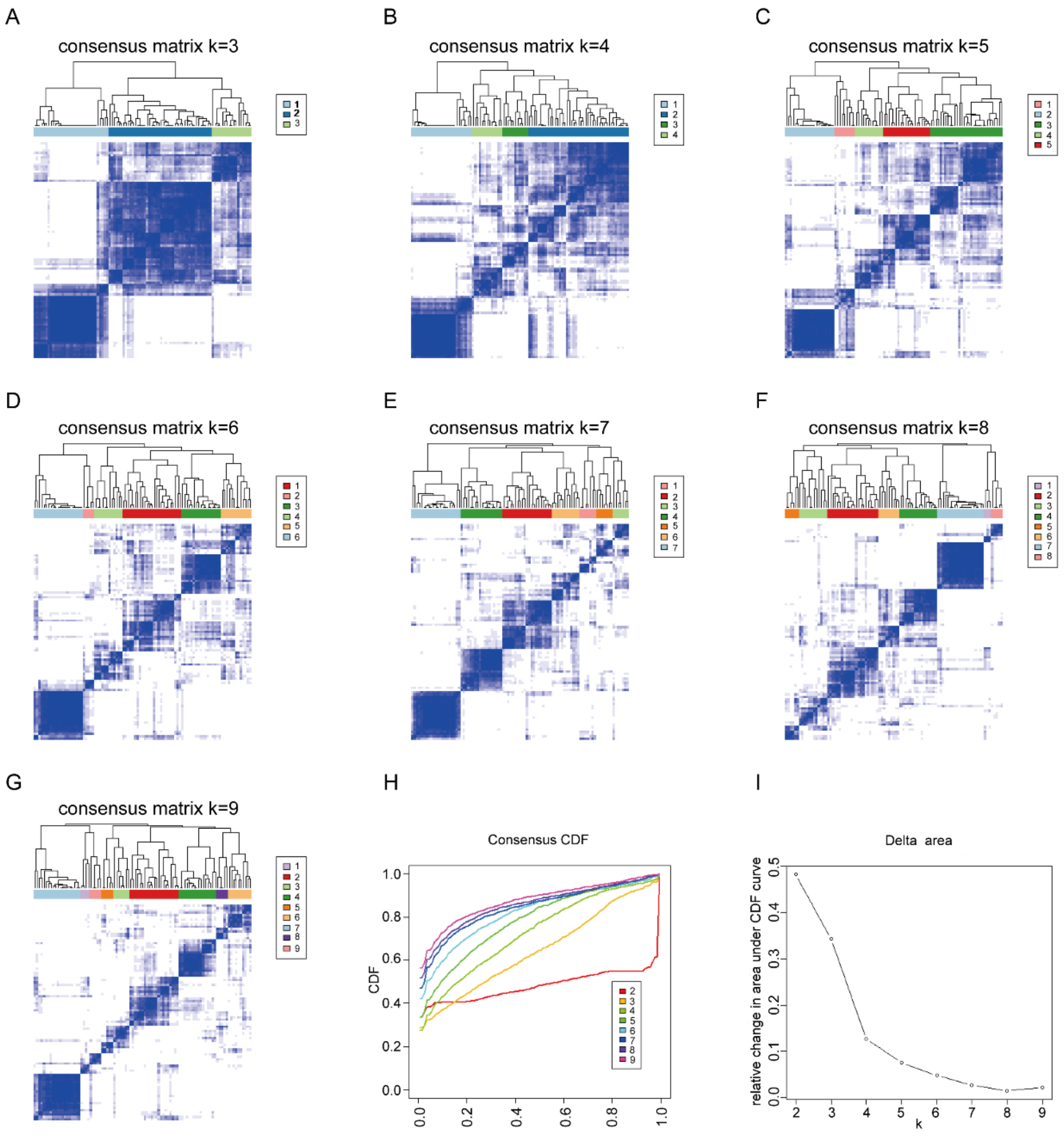

Supplementary Figure 2 (A-G) Consensus matrix heatmaps for k=3-9. **(H-I)** Consensus CDF curve and Delta area curve of consensus clustering analysis. K=2 was regarded as appropriate.

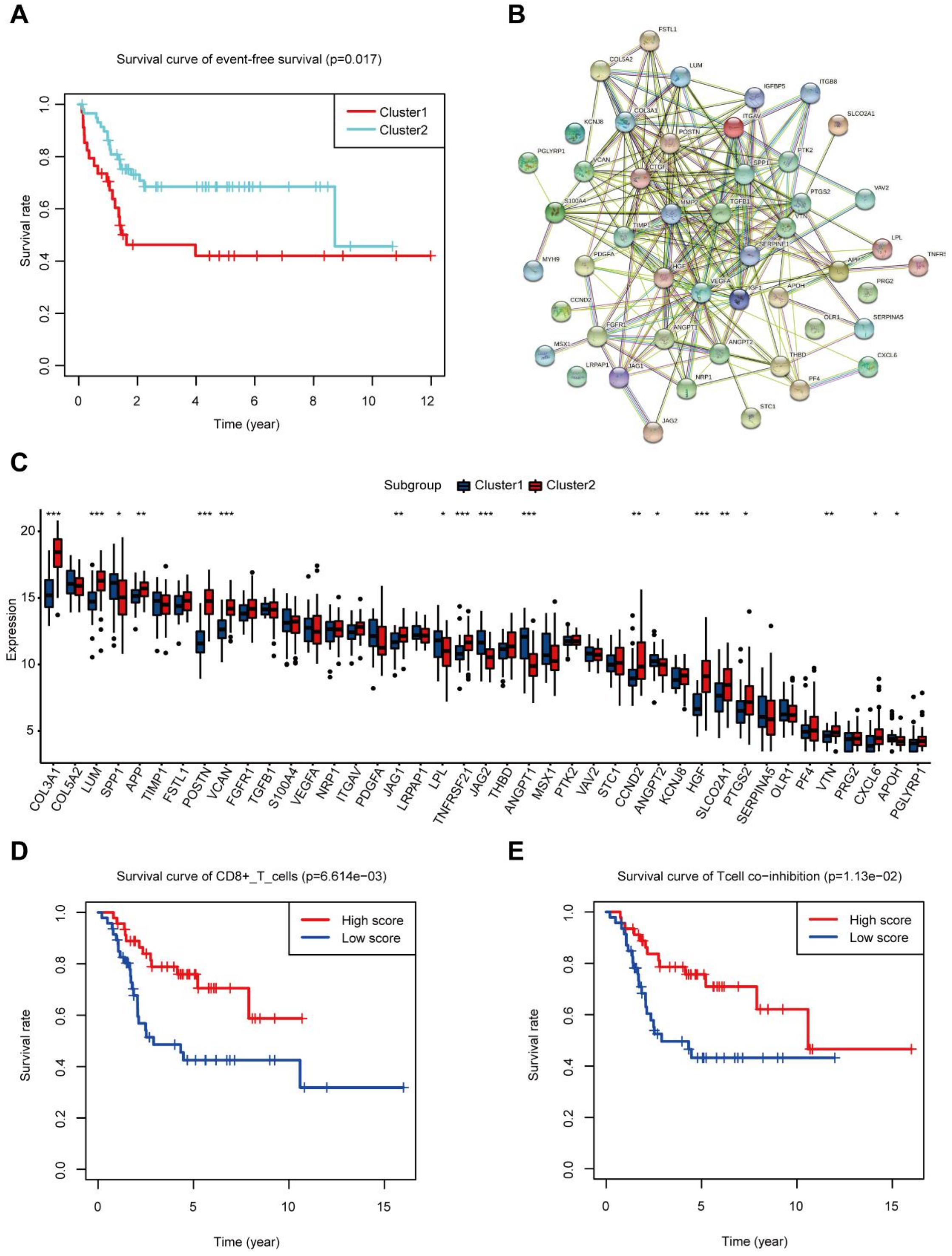

Supplementary Figure 3 (A) K-M plots comparing event-free survival between cluster1 and cluster2 in TARGET dataset (log-rank *p* value = 0.017). **(B)** Interactions of ARGs. **(C)** Boxplot of expression level of ARGs in two subgroups. **(D-E)** K-M plots comparing overall survival between low- and high- score group of CD8+ T cell and T cell co-inhibition, respectively (log-rank *p* value = 6.614e-3 and 1.13e-2). (**p* < 0.05, ***p* < 0.01, ****p* < 0.001).

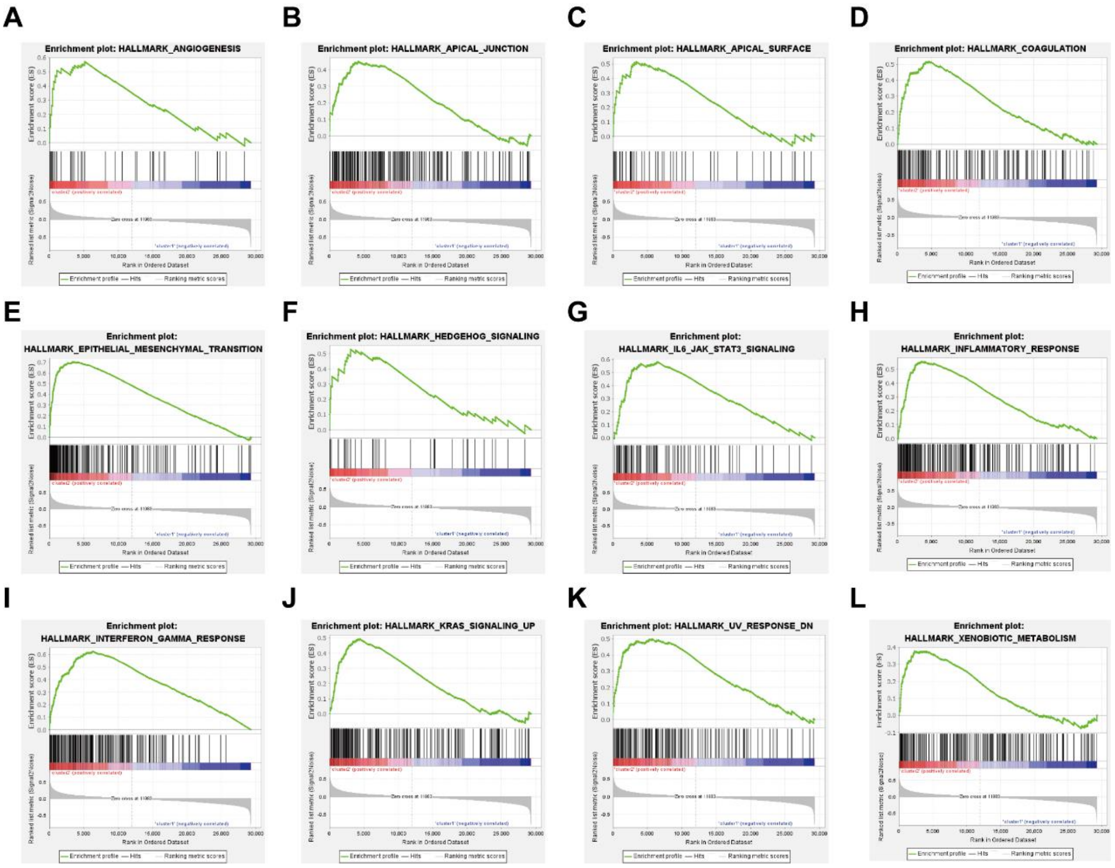

Supplementary Figure 4 (A-L) GSEA analysis of hallmark gene sets which were enriched in cluster2. *P* value < 0.05 and |normalized enrichment score (NES) | > 1.5 were deemed as significant enrichment.

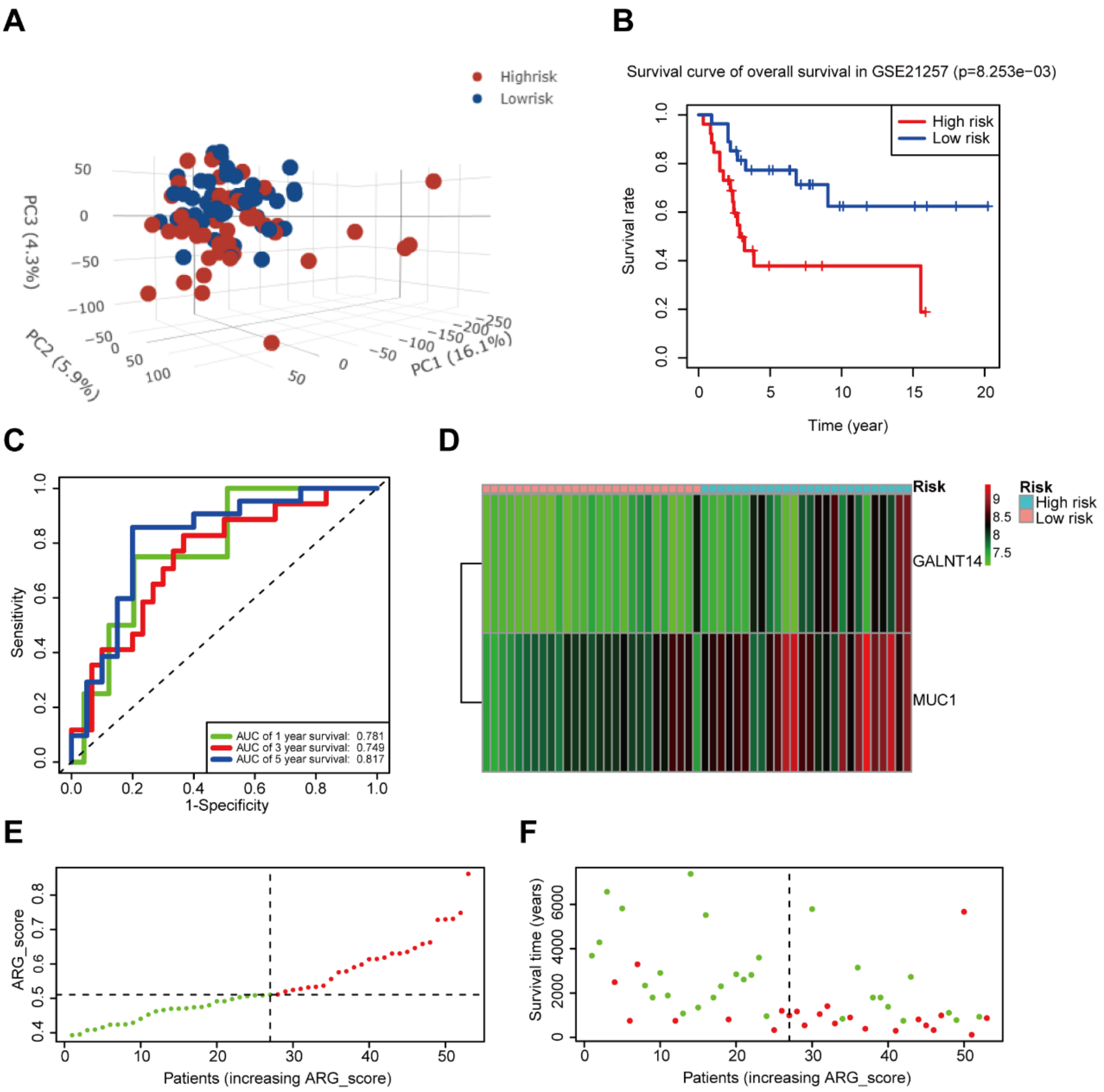

Supplementary Figure 5 (A) 3D PCA analysis of TARGET based on risk score model. **(B)** K-M plots comparing overall survival between low- and high-risk group in GSE21257 (log-rank *p* value = 8.253e-3). **(C)** Time-dependent ROC curves to predict the discrimination of 1-, 3-, 5-year survival according to risk score model in GSE21257. **(D)** A heatmap of two prognostic hub genes of GSE21257 in two subgroups. Red means high expression and green means low expression. **(E-F)** Ranked dot and scatter plots of GSE21257 showing the ARG_score distribution and patient survival status.

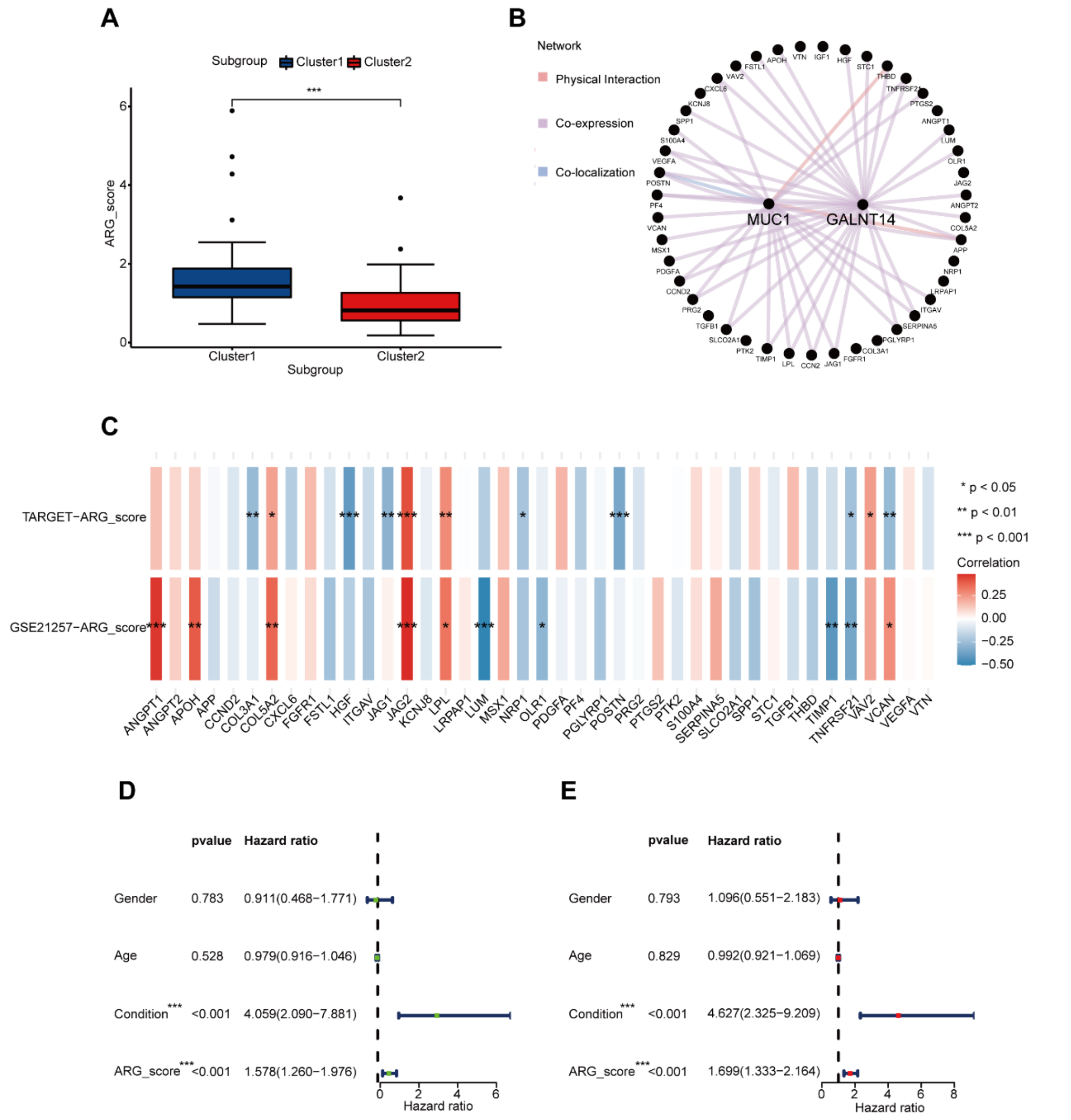

Supplementary Figure 6 (A) ARG_score in cluster1 and cluster2. **(B)** Interaction of ARGs and prognostic hub genes by GeneMANIA. **(C)** Correlation analysis between ARGs and ARG_score in TARGET and GSE21257. **(D-E)** Forest maps of univariate Cox regression and multivariate Cox regression. Hazard rate<1 was considered as protective factors while Hazard rate>1 was considered as risk factors. (*$p < 0.05$, **$p < 0.01$, ***$p < 0.001$).
